# ARL3 is downregulated and acts as a prognostic biomarker in glioma

**DOI:** 10.1186/s12967-019-1914-3

**Published:** 2019-06-24

**Authors:** Yulin Wang, Weijiang Zhao, Xin Liu, Gefei Guan, Minghua Zhuang

**Affiliations:** 1 Department of Neurosurgery, The First Affiliated Hospital of Shantou University Medical College, 57 Changping Road, Shantou, 515041 Guangdong China; 2 Center for Neuroscience, Shantou University Medical College, Shantou, 515041 Guangdong China; 3 Department of Stomatology, The First Affiliated Hospital of Shantou University Medical College, Shantou, 515041 Guangdong China; 4 Department of Neurosurgery, The First Hospital of China Medical University, Shenyang, 110001 Liaoning China

**Keywords:** ARL3, Glioma, Prognostic model, Angiogenesis, Tumor microenvironment

## Abstract

**Background:**

Glioma is the most common primary malignant brain tumor in adults with a poor prognosis. ARL3 is a member of the ARF family, and plays a key role in ciliary function and lipid-modified protein trafficking. ARL3 has been reported to be involved in ciliary diseases, in which it affects kidney and photoreceptor development. However, the functional role of ARL3 in cancer remains unknown. In this study, we aimed to explore ARL3 expression and its roles in glioma prognosis.

**Methods:**

RT-PCR and immunohistochemistry were performed to examine the expression level of ARL3 in glioma samples. Data from The Cancer Genome Atlas (TCGA), Chinese Glioma Genome Atlas (CGGA) and Repository for Molecular Brain Neoplasia Data (REMBRANDT) databases were employed to investigate ARL3 expression and its roles in glioma prognosis. A nomogram for predicting 3- or 5-year survival was established using Cox proportional hazards regression. Finally, gene ontology (GO) analysis, gene set enrichment analysis (GSEA), and gene set variation analysis (GSVA) were performed to explore the biological function.

**Results:**

ARL3 expression was downregulated in glioma, and associated with poor prognosis in glioma patients. The C-indexes, areas under the ROC curve and calibration plots of the nomogram indicated an effective predictive performance for glioma patients. In addition, GO and pathway analyses suggested the involvement of ARL3 in angiogenesis and immune cell infiltration in the microenvironment.

**Conclusions:**

Low ARL3 expression predicted poor prognosis and contributed to antiangiogenesis and the proportion of infiltrating immune cells in the GBM microenvironment. Thus, ARL3 may be a prognostic marker and therapeutic target for glioma.

**Electronic supplementary material:**

The online version of this article (10.1186/s12967-019-1914-3) contains supplementary material, which is available to authorized users.

## Introduction

Glioma is the most common primary intracranial tumor in adults and is notorious for its malignancy and unfavorable prognosis [1]. Despite standard treatment regimens, including surgery followed by radiation and chemotherapy, the prognosis of glioma patients is still dismal [2, 3]. Glioblastoma multiforme (GBM) is the most aggressive type, with a median survival between 14 and 18 months after diagnosis and an estimated 5-year survival rate of 5.1% [4]. Intratumoral heterogeneity of GBM is a key factor for the unsatisfactory therapeutic effect [5]. Heterogeneity in glioma could be affected by the tumor microenvironment, which provides a particular niche for glioma stem cells (GSCs) to promote glioma initiation, invasion, and therapeutic resistance [6]. Recently, several new therapeutic strategies, including oncogenic signal transduction inhibition/targeted therapy, antiangiogenesis, and immunotherapy, have attracted substantial attention and shed new light on the treatment of glioma [7–9].

ADP-ribosylation factor-like 3 (ARL3) is a kind of small GTP-binding protein in the ADP-ribosylation factor (ARF) family belonging to the RAS superfamily,  which is involved in multiple biological processes and tumor occurrence and progression [10–12]. ARF members regulate several essential cellular functions, such as membrane trafficking, cytoskeleton organization, and cell adhesion and migration, which are significantly relevant to tumor invasion and metastasis [13–16]. Current evidence has indicated that ARF proteins are involved in cancer progression through three different mechanisms: cell–cell adhesion, integrin trafficking and actin cytoskeleton rearrangement [14]. As a member of the ARF family, ARL3 has been reported to regulate cell morphology and cytokinesis through microtubule-based processes [17]. ARL3 interacts with dynactin and dynein to regulate microtubule mediated retrograde transport [18]. ARL3 can act as an allosteric release factor for farnesylated cargo and a regulator of trafficking of lipid-modified proteins [19, 20]. In addition, ARL3 influences ciliogenesis and is involved in ciliary function affecting kidney and photoreceptor development in mice [21, 22]. However, the specific functions of ARL3 in tumors remain unknown.

In this study, we investigated ARL3 expression and its roles in glioma prognosis using clinical samples and data from The Cancer Genome Atlas (TCGA), Chinese Glioma Genome Atlas (CGGA) and Repository for Molecular Brain Neoplasia Data (REMBRANDT) databases. Furthermore, a nomogram was constructed by applying the identified factors to predict 3- or 5-year survival for glioma patients. In addition, we explored the biological functions and pathways affected by ARL3 in glioblastoma, which may provide novel insights into glioma treatment.

## Materials and methods

### Patients and samples

Patient samples for PCR were collected at the First Hospital of China Medical University from February 2016 to June 2017. Nine glioma tissues (3 cases each of grade II, III and IV glioma) and 3 nontumor brain tissues from cranial injury internal decompression for control were included. The glioma and nontumor samples for immunohistochemistry staining and survival analysis (8 nontumor cases, 5 grade II cases, 17 grade III cases, and 24 grade IV cases) were collected at the First Hospital of China Medical University from January 2009 to June 2012. Histological diagnosis of the samples was confirmed by two neuropathologists according to the 2010 World Health Organization (WHO) classification guidelines. This study was approved by the Institutional Review Board of the First Hospital of China Medical University.

Gene expression and glioma patient survival data in the TCGA, REMBRANDT and Gravendeel databases were downloaded from GlioVis (http://gliovis.bioinfo.cnio.es/), and the CGGA date were obtained from http://www.cgga.org.cn/. RNA-seq data of 301 glioma patients with clinicopathologic characteristics from the CGGA were selected as the primary cohort to establish the predictive model and to construct the nomogram and risk classification system. A total of 211 cases from Gravendeel and 598 cases from TCGA were chosen as two independent validation cohorts. The inclusion criteria for data extraction in the predictive model were patients diagnosed with WHO grade II–IV glioma. The exclusion criteria included patients with missing or incomplete data such as survival status and time, age, sex, grade, and IDH status. Samples and immune infiltration data were downloaded from The Cancer Immunome Atlas (TCIA, https://tcia.at/home) and Tumor IMmune Estimation Resource (TIMER, https://cistrome.shinyapps.io/timer/) for immunogenomic analyses [23–25].

### RNA isolation and quantitative RT-PCR

Total RNA was isolated from clinical samples using TRIzol reagent (Invitrogen) according to the manufacturer’s protocol. Total RNA was reverse transcribed into cDNA and used for PCR amplification. Quantitative PCR was performed in a thermal cycler (Roche LightCycler 480) using *TransStart*^*®*^ Top Green qPCR SuperMix Assay (Transgen Biotech, AQ131). The following condictions were used for PCR: 1 cycle of 95 °C for 30 s, followed by 40 cycles of a two-step cycling program (95 °C for 5 s; 60 °C for 30 s). The mRNA expression was normalized to the expression of GAPDH mRNA and calculated by the 2^−ΔΔCt^ method. Specific primers for ARL3 and GAPDH were as follows: ARL3 forward 5′-GGACAGAGGAAAATCAGACCATACT-3′ and reverse 5′-GTCGCGGATGGTATGCAGGT-3′ [26] and GAPDH forward GAAGGTGAAGGTCGGAGTCA and reverse TTGAGGTCAATGAAGG GGTC [27].

### Immunohistochemistry (IHC)

Immunohistochemical procedures were performed as described previously [28]. In brief, after deparaffinization, antigen retrieval and endogenous peroxidase activity blocking, sections were blocked with normal goat serum for 15 min and incubated with anti-ARL3 primary antibody (Proteintech, 10961-AP, 1:250) at 4 °C overnight in a humidified chamber. After washing with PBS three times, the sections were incubated with biotinylated goat anti-rabbit IgG (ZSGB-BIO, SP-9001) for 15 min at room temperature. DAB was applied for staining after washing with PBS. The nuclei were counterstained with hematoxylin and the sections were mounted with coverslips after dehydration. Immunohistochemical staining was evaluated with a German immunohistochemical score (GIS) [29], which further classified the patients into low (GIS < 4) and high (GIS ≥ 4) ARL3 expression groups.

### Bioinformatics analysis

Genes related to ARL3 expression were extracted using Pearson’s correlation analysis (|r| ≥ 0.3). Gene ontology (GO) analysis was performed to analyze the related genes via AmiGO 2 (http://amigo.geneontology.org/amigo/landing) [30, 31] and DAVID website (https://david.ncifcrf.gov/) [32]. The gene set variation analysis (GSVA) package in R was used to explore biological processes and KEGG pathways between low and high ARL3 expression groups [33]. Gene terms with |logFC| ≥ 0.2 and P < 0.05 were considered statistically significant. Venn diagrams, bar charts and heatmaps were mapped using R version 3.5.1. Gene set enrichment analysis (GSEA, http://software.broadinstitute.org/gsea/index.jsp) was employed to verify the biological processes in the two groups stratified as described above [34]. Normalized enrichment score (NES) and false discovery rate (FDR) were calculated to verify the significant difference for GSEA. Cytoscape 3.5.1 version with ClueGO was used to search and visualize signal pathways in KEGG and Reactome for the related gene sets. The pathways with P < 0.01 were visualized in Cytoscape. The locations of ARL3 in glioblastoma anatomic structures were analyzed by the Ivy Glioblastoma Atlas Project (IVY GAP, http://glioblastoma.alleninstitute.org/static/home) [35, 36].

### Statistical analysis

Quantitative data are presented as the mean ± standard derivation. Statistical differences between and among groups were examined by two-tailed t-tests and one-way analysis of variance (ANOVA) followed by Dunnett’s posttest, respectively. The survival data were analyzed with Kaplan–Meier curves and log-rank tests. The statistical analysis of ARL3 expression and survival data were performed using GraphPad Prism 7.0 and Excel 2013. Univariate and multivariate Cox proportional hazards models were established using R version 3.5.1. P < 0.05 was considered statistically significant. The nomogram and risk classification system were constructed using the rms package and predict function respectively in R and validated by two independent cohorts from TCGA and Gravendeel databases. The performance of the nomogram was measured by concordance index (C-index), ROC curve and calibration curve established in R.

## Results

### ARL3 is expressed at low levels in glioma

**Fig. 1:**
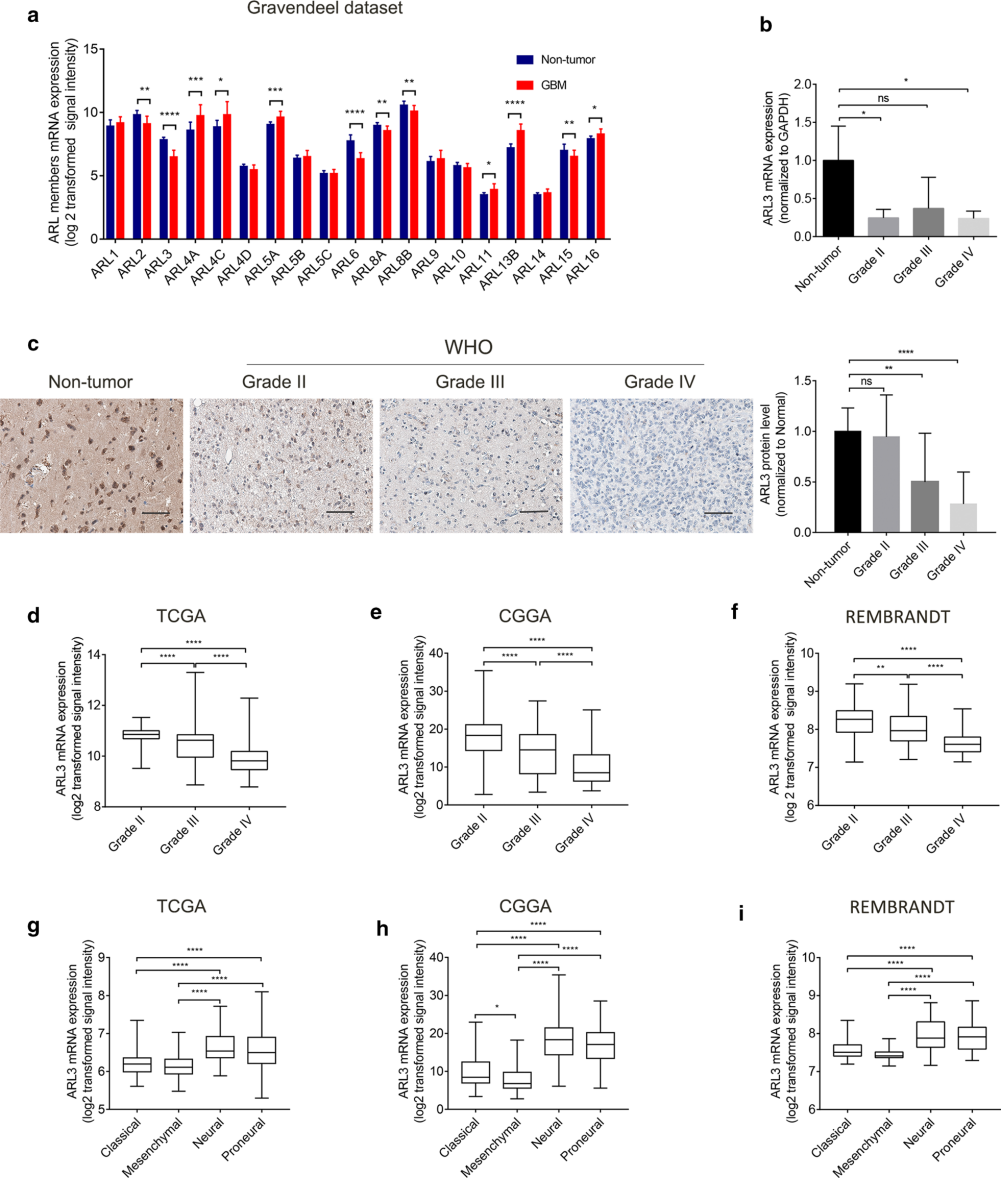
Expression of ARL3 was decreased in GBM. **a** Data from the Gravendeel database showed the differential expression of nineteen ARL family members between nontumor tissues and GBM samples. **b** qRT-PCR analyses of ARL3 mRNA in glioma (WHO grade II–IV) and nontumor brain tissues (nontumor, n = 3; grade II, n = 3; grade III, n = 3; grade IV, n = 3) (*P < 0.05, with one-way ANOVA). **c** Immunohistochemical staining of ARL3 in glioma (WHO grade II–IV) and nontumor tissues (nontumor, n = 8; grade II, n = 5; grade III, n = 17; grade IV, n = 24) (**P < 0.01, ****P < 0.0001, with one-way ANOVA). Scale bar, 50 μm. **d** Data from TCGA (RNA-seq; grade II, n = 226; grade III, n = 244; grade IV, n = 150) revealed that ARL3 expression decreased in GBM compared with that in grade II and III glioma (****P < 0.0001, with one-way ANOVA). **e** Data from the CGGA (RNA-seq; grade II, n = 109; grade III, n = 72; grade IV, n = 144) showed that ARL3 was downregulated in GBM compared with that in grade II and III glioma (****P < 0.0001, with one-way ANOVA). **f** Data from REMBRANDT (Microarray; grade II, n = 98; grade III, n = 85; grade IV, n = 130) revealed that ARL3 expression in GBM was lower than that in grade II and III glioma (**P < 0.01, ****P < 0.0001, with one-way ANOVA). **g** Data from TCGA showed that ARL3 was downregulated in the mesenchymal subtype compared with that in neural and proneural subtypes (RNA-seq; classical, n = 145; mesenchymal, n = 157; neural, n = 88; proneural, n = 138) (****P < 0.0001, with one-way ANOVA). **h** The level of ARL3 expression was significantly decreased in the mesenchymal subtype of glioma compared with that in the other three subtypes (CGGA, RNA-seq; classical, n = 74; mesenchymal, n = 68; neural, n = 81; proneural, n = 102) (*P < 0.05, ****P < 0.0001, with one-way ANOVA). **i** Data from Rembrandt showed ARL3 expression was decreased in the mesenchymal subtype compared with that in neural and proneural subtypes (RNA-seq; classical, n = 99; mesenchymal, n = 37; neural, n = 44; proneural, n = 39) (****P < 0.0001, with one-way ANOVA)

Although a previous study has shown that ARL2 inhibits glioma proliferation and tumorigenicity by downregulating AXL [28], the functions of other ARL members in glioma remained unknown. We collected expression and survival data of 19 members of the ARL subfamily from the Gravendeel database and confirmed 12 differentially expressed genes between nontumor and GBM samples (Fig. 1a). Univariate Cox analyses were performed, and 4 genes (ARL3, ARL4A, ARL4C and ARL11) were found to be associated with the prognosis of patients with GBM (Additional file 1: Table S1). It has been reported that human ARL3 and ARL2 share 53% similarity in primary sequence and interact with the same set of effectors, such as BART and PDE6δ [17, 20]. Therefore, ARL3 was chosen for further exploration in glioma.

Twelve human clinical samples, including 9 glioma tissues (3 cases each of grade II–IV) and 3 nontumor brain tissues, were collected. Quantitative PCR was performed on these specimens. The results revealed that ARL3 mRNA levels decreased in both grade IV (GBM) and grade II samples in comparison with those in nontumor tissues (Fig. 1b). Then, 46 clinical glioma samples were used to examine ARL3 expression in glioma by IHC. The results confirmed that ARL3 expression was downregulated in grade IV and grade III glioma samples (Fig. 1c). Moreover, we investigated the level of ARL3 expression in multiple datasets to confirm the above results. As expected, the data for ARL3 expression in TCGA was consistent with previous results and reduced ARL3 mRNA expression was detected in GBM tissues compared with that in grade II and III tissues (Fig. 1d). ARL3 expression data in the CGGA and REMBRANDT databases also confirmed lower levels of ARL3 expression in GBM samples than those in grades II and III samples (Fig. 1e, f). To extend these observations, we examined ARL3 expression in different subtypes of glioma in the TCGA, CGGA and REMBRANDT datasets. The data showed that mesenchymal subtype had the lowest expression level of ARL3 among the four subtypes (Fig. 1g–i). Collectively, ARL3 is downregulated in GBM.

### Low expression of ARL3 indicates poor prognosis of glioma patients

**Fig. 2:**
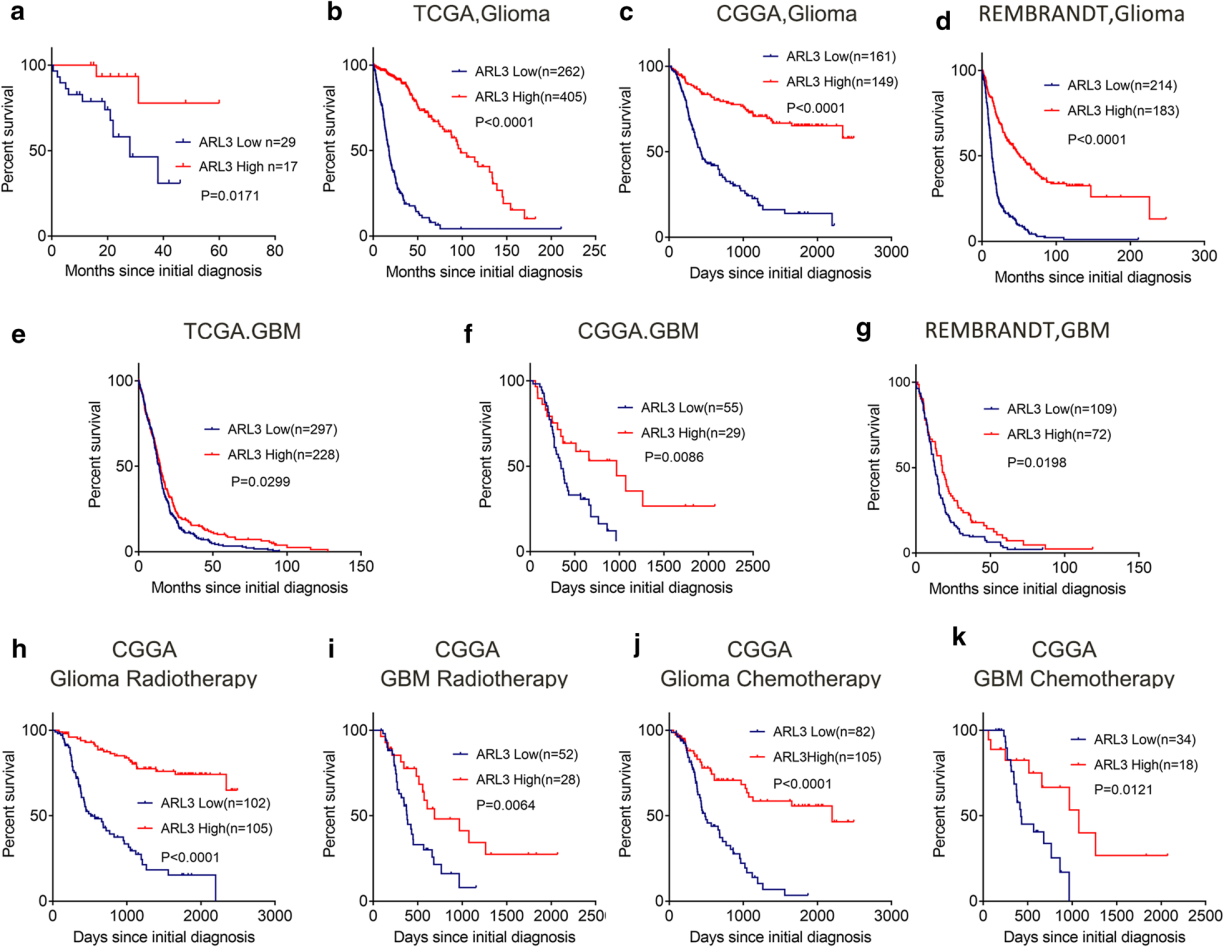
ARL3 expression level was associated with the prognosis of glioma patients and the response to chemo- and radio-therapy. **a** Kaplan–Meier analyses to evaluate the correlation between ARL3 expression and the survival of glioma patients (low, n = 29; high, n = 17; P = 0.0171, with the log-rank test). Data from the TCGA (**b**, RNA-seq; low, n = 262; high, n = 405; *P* < 0.0001, with the log-rank test), CGGA (**c**, RNA-seq; low, n = 161; high, n = 149; *P* < 0.0001, with the log-rank test) and REMBRANDT (**d**, microarray; low, n = 214; high, n = 183; *P* < 0.0001, with the log-rank test) datasets indicated that lower ARL3 expression was correlated to poorer prognosis of glioma patients. **e**–**g** Data from the TCGA (**b**, RNA-seq; low, n = 297; high, n = 228; *P* = 0.0299, with the log-rank test), CGGA (**c**, RNA-seq; low, n = 55; high, n = 29; *P* = 0.0086, with the log-rank test) and REMBRANDT (**d**, microarray; low, n = 109; high, n = 72; *P* = 0.0198, with the log-rank test) datasets indicated that reduced ARL3 expression was associated with unfavorable prognosis in GBM patients. **h** Kaplan–Meier analysis of survival of glioma patients treated with radiotherapy from CGGA (RNA-seq, low, n = 102; high, n = 105; *P* < 0.0001, with log-rank test). **i** Kaplan–Meier analysis of the survival of GBM patients treated with radiotherapy from the CGGA according to ARL3 expression (RNA-seq, low, n = 52; high, n = 28; *P* = 0.0064, with the log-rank test). **j** Kaplan–Meier analysis of the survival of glioma patients treated with chemotherapy from the CGGA according to ARL3 expression (RNA-seq, low, n = 82; high, n = 105; *P* < 0.0001, with the log-rank test). **k** Kaplan–Meier analysis of the survival of primary GBM patients treated with chemotherapy from the CGGA (RNA-seq, low, n = 34; high, n = 18; *P* = 0.0121, with the log-rank test)

To explore the prognostic value of ARL3, we collected survival data from 46 patients with different grades of glioma (shown in Additional file 2: Table S2) and investigated the relationship between ARL3 expression level and prognosis. The results demonstrated that low ARL3 expression was negatively correlated with glioma patient outcome (Fig. 2a). To further confirm this result, we downloaded survival data from the TCGA, CGGA and REMBRANDT databases and performed survival analysis to investigate the clinical relevance of ARL3 expression in patient survival. The results demonstrated that elevated ARL3 expression was clinically correlated with favorable outcomes of glioma patients (Fig. 2b–d). Similar results were also obtained from the patients with GBM, showing that lower ARL3 expression resulted in a poorer patient prognosis than higher ARL3 expression (Fig. 2e–g). Taken together, these results suggest the potential value of ARL3 as a marker in the outcome prediction of glioma patients.

### ARL3 expression suggests differential responses to radiation and chemotherapy

Since the application of standard radio- and chemo-therapy in the treatment of malignant glioma has been well established, we further analyzed the association between ARL3 expression and the response to standard radio- and chemo-therapy according to the CGGA and TCGA datasets. Samples were divided into low and high expression groups based on the mean level of ARL3 mRNA expression. The results in the CGGA revealed that the glioma patients receiving radiotherapy in the high ARL3 expression group had a better prognosis than patients in the low ARL3 expression group (Fig. 2i). In patients with GBM with high ARL3 expression, a better curative effect of radiotherapy was observed than in patients with low ARL3 expression (Fig. 2j). In terms of chemotherapy, the survival time in the high ARL3 expression group was longer than that in the low ARL3 expression group among glioma patients as well as in primary GBM patients (Fig. 2k, l). The observations were verified by data in TCGA, indicating that GBM patients receiving radiation or chemotherapy in the low ARL3 expression group had a poorer prognosis than patients in the high expression group (Additional file 3: Fig. S1a, b). These findings further confirmed that ARL3 may function as a biomarker for predicting the response to radio- and chemo-therapy in glioma patients.

### ARL3-related prognostic nomogram

In view of the prognostic value of ARL3 in glioma, we constructed a nomogram and risk classification system for predicting 3- and 5-year survival. In the primary cohort, 301 glioma cases from the CGGA were included. A total of 211 cases from the Gravendeel and 598 cases from the TCGA were chosen as two independent validation cohorts. The demographic and clinicopathologic characteristics of patients in the primary and validation cohorts are listed in Table 1. A Cox proportional hazards model was employed in the primary cohort to assess the value of each variable in predicting the prognosis of glioma patients. Univariate and multivariate analyses indicated that factors such as ARL3 expression level and WHO grade were significantly correlated with patient prognosis (Table 2). The criteria for selecting variables conformed to clinical relevance and multivariate Cox analysis [37]. It has been reported that age, IDH status and sex are associated with the incidence rate or prognosis of glioma [1, 38–40]. Considering the clinical factors of glioma, these parameters (ARL3 expression level, age, sex, WHO grade and IDH status) were included in the predictive model.

**Table 1:** Demographics and clinicopathologic characteristics of patients with glioma

Characteristic	Primary cohort		Validation cohort			
	CGGA (n = 310)		Gravendeel (n = 211)		TCGA (n = 598)	
	No. of patients	%	No. of patients	%	No. of patients	%
ARL3 level						
Median	13.402		6.658		10.623	
Range	2.762–28.522		5.591–8.921		8.787–13.299	
Gender						
Male	195	62.903	146	69.194	350	58.528
Female	115	37.097	65	30.806	248	41.472
Age, years						
Median	43		50		47	
Range	8–81		14–81		14–89	
WHO grade						
Grade II	105	33.871	21	9.953	211	35.284
Grade III	67	21.613	66	31.280	239	39.967
Grade IV	138	44.516	124	58.768	148	24.749
IDH status						
Wild type	146	47.097	131	62.085	222	37.124
Mutant	164	52.903	80	37.915	376	62.876

**Table 2:** Univariate analysis and multivariate analysis of overall survival in the primary cohort (CGGA)

Variables	Univariate analysis			Multivariate analysis		
	P value	HR	95% CI	P value	HR	95% CI
ARL3 level	< 0.0001	0.873	0.845–0.901	0.0095	0.949	0.913–0.987
Gender						
Male	Reference			Reference		
Female	0.345	0.847	0.60–1.195	0.7251	0.951	0.717–1.261
Age	< 0.0001	1.038	1.023–1.054	0.7727	1.002	0.987–1.018
WHO grade						
Grade II	Reference			Reference		
Grade III	< 0.0001	5.862	3.185–10.79	< 0.0001	4.508	2.395–8.486
Grade IV	< 0.0001	14.707	8.276–26.13	< 0.0001	8.658	4.578–16.377
IDH status						
Wild type	Reference			Reference		
Mutant	< 0.0001	0.244	0.17–0.35	0.1475	0.705	0.439–1.132

**Fig. 3:**
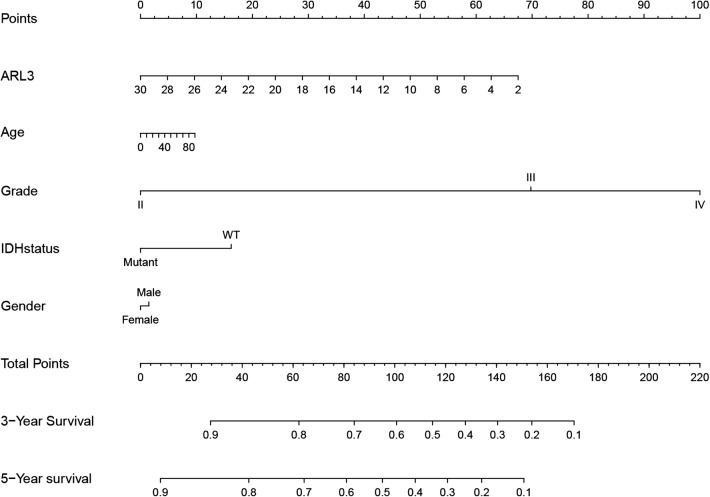
Nomogram for predicting 3 or 5-year survival in glioma patients. The top row shows the point value for each variable. Rows 2–6 indicate the variables included in the nomogram. Each variable corresponds to a point value based on glioma characteristics. The sum of these values is located on the Total Points axis, and the line drawn downward to the survival axes is used to determine the likelihood of 3- or 5-year survival. ARL3 was represented as the mRNA expression level in RNA-seq with log2 transformation

**Fig. 4:**
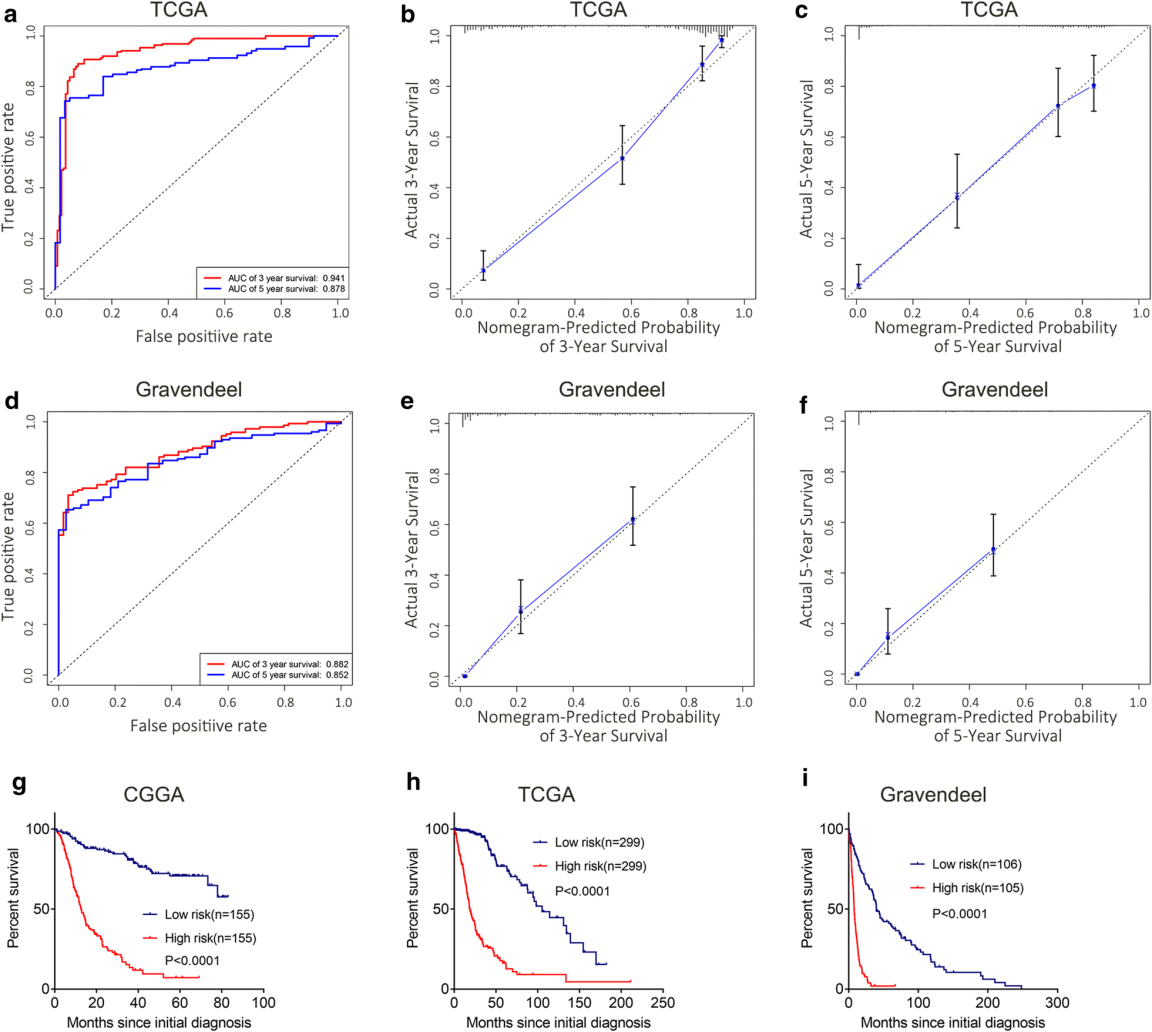
Evaluation of the nomogram and risk classification system for predicting 3- and 5-year survival. **a** Receiver operating characteristic (ROC) curve was used for discrimination of 3- or 5-year survival in the validation cohort (TCGA). The areas under the curves (AUCs) for the nomogram were 0.941 and 0.878 respectively, suggesting a very good predictive performance. Calibration curves for predicting patient survival at 3 years (**b**) and 5 years (**c**) in the validation cohort (TCGA). Nomogram-predicted probability of survival was plotted on the x-axis; actual survival was plotted on the y-axis. **d** The AUCs of the nomogram for predicting 3- and 5-years survival were 0.882 and 0.852, respectively, suggesting good predictive performances in the validation set (Gravendeel). **e**, **f** Calibration plots for the probability of survival at 3-year and 5-year showed an optimal agreement between the prediction and observation in the validation cohort (Gravendeel). Kaplan–Meier analysis of survival between the low-risk and high-risk groups according to the risk classification system in the primary cohort (**g**, CGGA) and the validation cohort (**h**, TCGA and **i**, Gravendeel)

The predictive model was presented as a nomogram and is shown in Fig. 3. The C-index of the nomogram was 0.764 in the primary cohort, 0.729 in the Gravendeel cohort and 0.859 in the TCGA cohort. A receiver operating characteristic (ROC) curve was used to evaluate the accuracy of prediction of 3- and 5-year survival in the primary and validation sets. The area under the curve (AUC) of the nomogram for 3-year survival was 0.932 in the primary cohort, 0.941 in the TCGA cohort and 0.882 in the Gravendeel cohort, and the AUC of 5-year survival in the nomogram were 0.898 in the primary cohort, 0.878 in the TCGA cohort and 0.852 in the Gravendeel cohort (Additional file 3: Fig. S2a and Fig. 4a, d). The calibration plot for the probability of survival at 3 or 5 years showed an optimal agreement between the prediction and observation in the primary cohort (Additional file 3: Fig. S2b, c), as well as in the validation cohort (Fig. 4b, c, e, f). These nomogram-based results demonstrated a good accuracy for predicting the 3- or 5-year survival of glioma patients.

In addition, a risk classification system for predicting the prognosis of glioma patients was developed. Patients in each cohort were divided into low-risk and high-risk groups according to the median cutoff value of the risk scores. The Kaplan–Meier curves showed that the high-risk group exhibited poorer prognosis than the low-risk group in both the primary cohort and the validation cohorts (Fig. 4e–g). These data suggested that ARL3 is an independent prognostic factor that can be used to competently predict the survival of patients with glioma.

### ARL3-related biological signatures in GBM

**Fig. 5:**
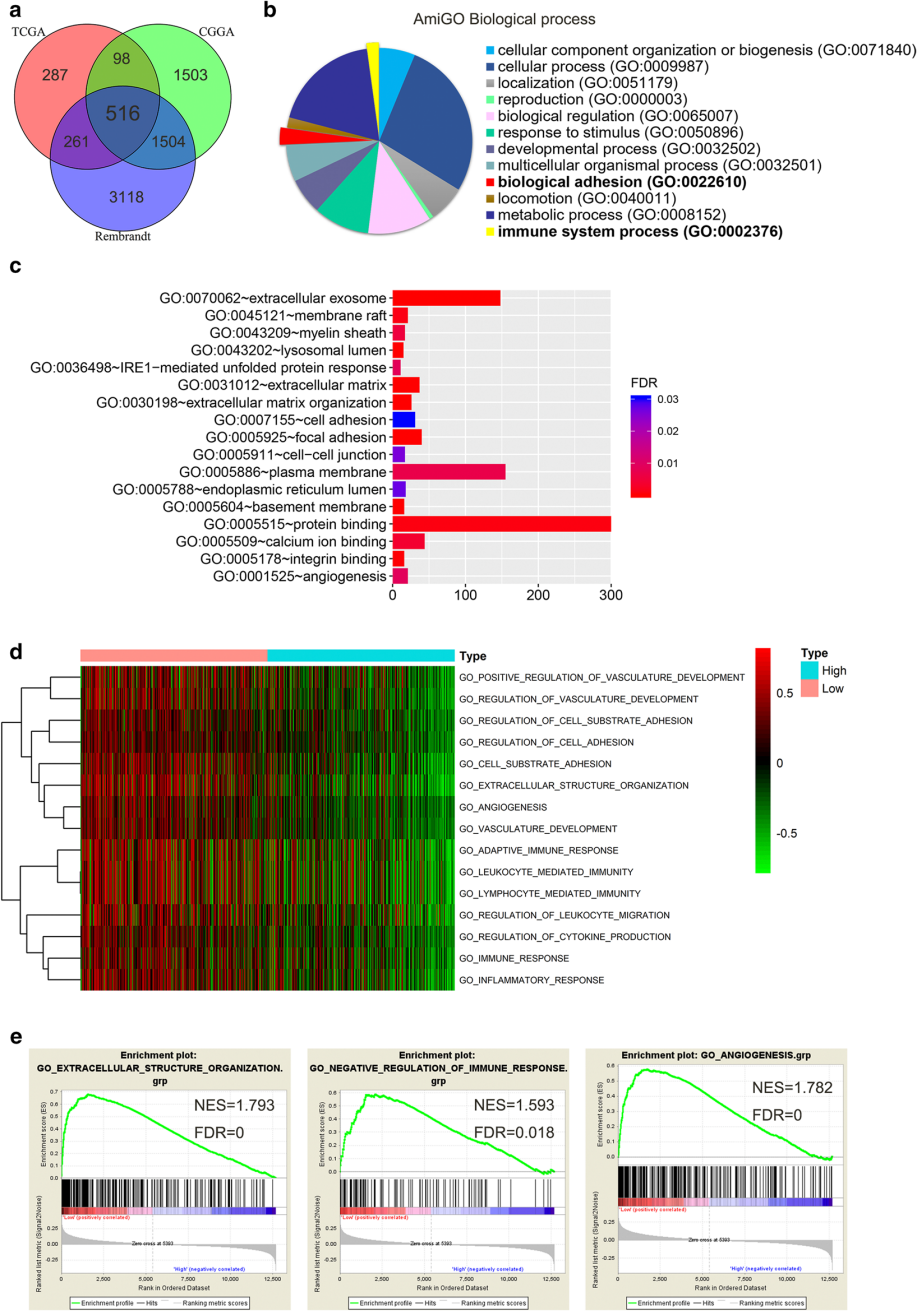
ARL3-related biological signatures in GBM. **a** Related genes of ARL3 were chosen in GBM from the TCGA, CGGA and REMBRANDT datasets based on Pearson’s correlation analysis (|r| ≥ 0.3), and 516 intersection genes were screened out. **b** ARL3-related biological processes in GBM via AmiGO2 (http://amigo.geneontology.org/amigo/landing). **c** ARL3-related GO terms in GBM via DAVID (https://david.ncifcrf.gov/). **d** GSVA was applied to analyze the overlapping genes in TCGA and representative terms are listed. **e** GSEA used to validate the gene signatures, including extracellular matrix organization, negative regulation of immune response and angiogenesis phenotype

To elucidate the function of ARL3 in GBM, we first searched for genes correlated with ARL3 expression in GBM through Pearson’s correlation analysis (|r| ≥ 0.3) in the TCGA, CGGA and REMBRANDT datasets. A total of 516 genes were found in the intersection of the three datasets (Fig. 5a, Additional file 4: Table S3). Gene ontology analysis was performed to evaluate the related 516 genes via AmiGO2 and DAVID. We discovered that ARL3 was functionally associated with multiple biological processes including biological adhesion, immune regulation, extracellular matrix and angiogenesis (Fig. 5b, c). Similar conclusions were also obtained from GSVA (Additional file 5: Table S4), and several representative terms are listed in Fig. 5d. In addition, GSEA was performed, and the results confirmed the gene signatures, including extracellular matrix organization, immune response and angiogenesis phenotype (Fig. 5e). Tumor microenvironment consists of tumor cells, infiltrated immune cells, stromal cells, extracellular matrix (ECM) and chemical factors, and is recognized as a key factor in tumor progression [41, 42]. Likewise, glioma cells attach and remodel the microenvironment by releasing extracellular signal molecules that promote angiogenesis, ECM remodeling, and immune escape [43]. In view of the biological processes of ARL3 in GBM, we concluded that ARL3 plays an important role in the glioma immune microenvironment and angiogenesis.

### ARL3-related signaling pathways in GBM

**Fig. 6:**
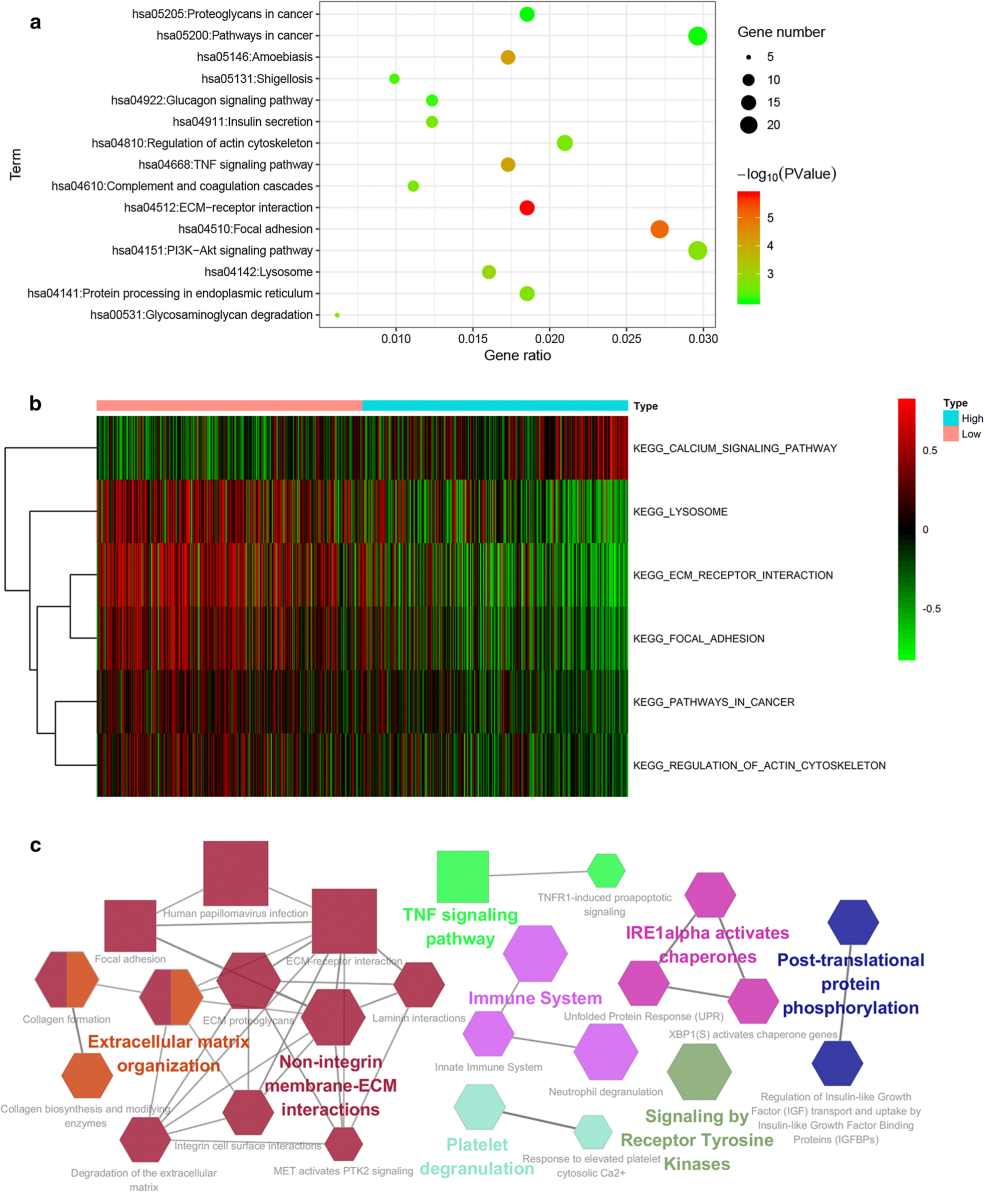
ARL3-related signal pathways in GBM. ARL3-related KEGG pathways via DAVID (**a**) and GSVA in TCGA (**b**) showing that ARL3 was involved in pathways in cancer, focal adhesion and ECM receptor interaction. **c** ARL3-related pathways in KEGG (rectangle) and Reactome (hexagon) are visualized using Cytoscape

To better understand the function role of ARL3 in GBM, we conducted KEGG pathway analysis for the 516 related genes via DAVID and GSVA. The results from DAVID showed that ARL3 was involved in pathways in cancer, focal adhesion, ECM receptor interaction and leukocyte transendothelial migration (Fig. 6a). Similar conclusions were confirmed by GSVA, and 5 GO terms that were positively correlated with low ARL3 expression (Fig. 6b). Cytoscape was also employed to analyze ARL3-related genes and display the interaction among pathways. The Cytoscape-based results illustrated enriched terms centrally attached to extracellular matrix-related pathways (Fig. 6c). Collectively, these data indicated that ARL3 is closely correlated with extracellular matrix-related pathways.

### ARL3 negatively regulates angiogenesis in GBM

**Fig. 7:**
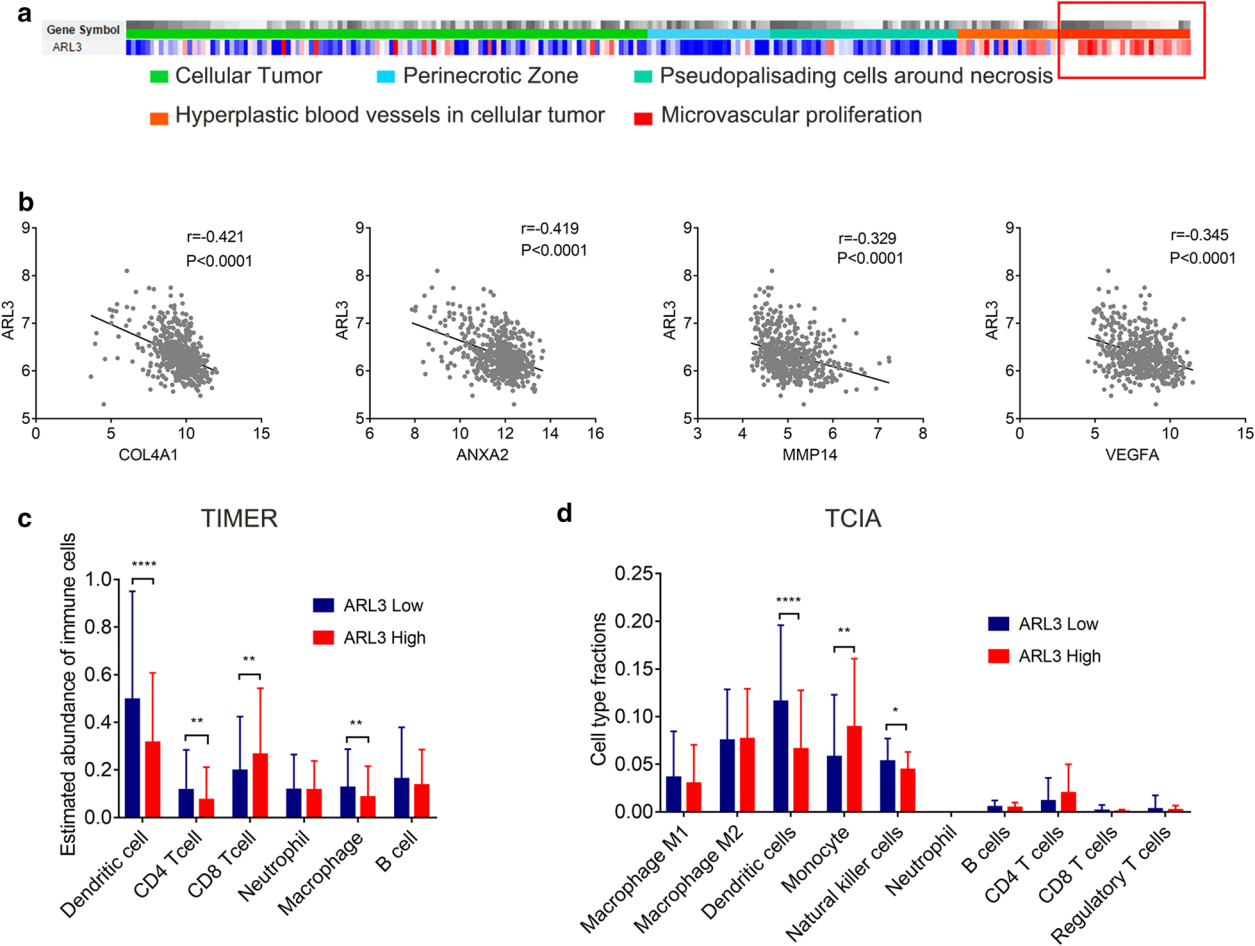
ARL3 negatively regulated angiogenesis and influenced infiltrating immune cells in GBM. **a** IVY GAP (http://glioblastoma.alleninstitute.org/static/home) was employed to analyze the locations of ARL3 in glioblastoma anatomic structures. **b** Pearson’s correlation analysis showed that ARL3 was negatively correlated with several proangiogenic genes (COL4A1, ANXA2, VEGFA, MMP14) in TCGA (HG-UG133A). Data from TIMER (**c**) and TCIA (**d**) indicated that ARL3 influenced the proportion of infiltrating immune cells in GBM

To validate the conclusion that ARL3 is involved in angiogenesis in GBM, we analyzed the expression of ARL3 in the RNA-seq database of the Ivy Glioblastoma Atlas Project. The RNA-seq profiles contain GBM samples from different laser-microdissected structures, including cellular tumor, perinecrotic zone, pseudopalisading cells around necrosis, hyperplastic blood vessels in cellular tumor and proliferating microvasculature. As shown in Fig. 7a, ARL3 was highly expressed in the proliferating microvascular area of GBM. In addition, we determined the correlation between ARL3 and several proangiogenic genes (COL4A1, ANXA2, VEGFA, MMP14) [44–46] via Pearson’s correlation analysis. The data showed that ARL3 was negatively correlated with these proangiogenic genes (Fig. 7b). These results indicated that ARL3 negatively regulates angiogenesis in GBM.

### ARL3 influences the proportion of infiltrating immune cells in GBM

Tumor-infiltrating immune cells are a part of a complex microenvironment that regulates tumor development and progression [47]. Since GO analysis revealed that ARL3 was related to the immune response, we further explored the infiltration of immune cells in GBM. Data were downloaded from TIMER and TICA, and subdivided into low and high groups according to the expression level of ARL3. The data from TIMER showed that the samples with low ARL3 expression had a relatively higher abundance of infiltrating dendritic cells, CD4 T cells and macrophages, but a lower abundance of CD8 T cells (Fig. 7c). In TCIA, samples with low ARL3 expression contained a relatively higher percentage of dendritic cells and NK cells and a lower percentage of monocytes than samples with high ARL3 expression (Fig. 7d). Taken together, these results indicated that ARL3 influences the infiltration of immune cells into the glioblastoma microenvironment.

## Discussion

Presently, treatment of glioblastoma is still an enormous challenge due to its aggressiveness and high rate of recurrence [48]. Although biotechnology and several innovative approaches have been adopted, no progress has been made in progression-free survival (PFS) and overall survival (OS) in GBM patients [49]. Intratumor heterogeneity is one of the most important hallmarks of GBM, which gives rise to therapeutic resistance and tumor recurrence [50]. It is thus urgent to precisely evaluate the prognosis of GBM patients and apply personalized treatment strategies.

For a more accurate prognostic prediction, nomograms have been developed, and these nomograms show better performance than conventional staging systems in some cancers [51, 52]. In this study, we identified ARL3 as a prognostic marker for glioma, and constructed a nomogram and risk classification system. The nomogram included five parameters that are readily available from clinical records and tissue specimens. As reported previously, age is an independent prognostic factor, and older ages are associated with poorer prognosis [53]. For sex, males have a higher incidence of GBM than females [54]. IDH mutation is an early event in gliomagenesis and is implicated in glioma progression [55]. Wild-type IDH and higher WHO grade (III or IV) have been proven to be associated with adverse outcomes [56]. These results are consistent with our findings. In the validation cohorts, the C-indexes, areas under the ROC curve (all above 0.85) and highly fitted calibration plots demonstrated that the nomogram performed well in predicting 3- or 5-year survival for patients with glioma. However, there are some limitations: first, the sample size used in the nomogram was small; second, the primary and validation cohorts were collected from datasets, and they did not contain details of intervention, such as extent of glioma resection, radiotherapy and chemotherapy. In future studies, we may incorporate detailed clinical records and apply the nomogram into clinical practice.

Another novel finding of this study is that ARL3 is involved in the glioma immune microenvironment and angiogenesis. Angiogenesis has been reported to contribute to glioma growth, invasion and metastasis, and increased tumor microvascular density (MVD) indicates poor prognosis [57]. Several proangiogenic factors secreted by tumor cells, stromal cells and inflammatory cells in the tumor microenvironment promote angiogenesis [45, 58]. The proangiogenic factors include VEGF family proteins (including VEGFA, VEGFB, VEGFC and VEGFD) and placental growth factors, and serve as treatment targets [57]. Although bevacizumab, an anti-VEGFA antibody, has shown efficacy by prolonging PFS in clinical trials for glioblastoma, it fails to affect overall survival [59]. The reason for the lack of benefits on OS is ascribed to the use of the drug for an unselected patient population; thus, it is necessary to identify biomarkers to predict the response of antiangiogenic agents [59, 60]. Using bioinformatics analyses, our study revealed that ARL3 was closely correlated with angiogenesis. More importantly, we found that ARL3 was highly expressed in proliferating microvascular area of GBM and negatively correlated with proangiogenic genes, such as VEGFA. Overall, these results suggested that ARL3 negatively regulates angiogenesis and represents a potential target for antiangiogenic therapy in GBM.

In addition, angiogenesis plays an important role in the immune composition of the tumor microenvironment [61]. A recent study revealed that antiangiogenesis therapy increases the abundance of mature DCs and enhances CD8 T cell immunity against glioma [9]. Here, we also observed that ARL3 influences the infiltration of immune cells in the glioblastoma microenvironment. Samples with low ARL3 expression tended to harbor a higher proportion of dendritic cells (DCs), macrophages, NK cells, CD4 T cells and a lower proportion of CD8 T cells. Infiltrating immune cells are important components of the tumor microenvironment and are associated with tumor behavior and patient outcomes. Glioma cells release multiple cytokines, interleukins and growth factors that promote the infiltration of various cells, including astrocytes, pericytes, endothelial cells, circulating progenitor cells, and immune cells such as microglia, peripheral macrophages, myeloid-derived suppressor cells, leukocytes, CD4 T cells, and Tregs into the tumor [62]. Glioma not only recruits immune cells, but also modifies them to evade immune surveillance. In a mouse glioma model, it has been found that DCs downregulate costimulatory molecules (CD40, B7.1, B7.2) and are unable to stimulate T cells [47]. Another study observed that glioma cells induce abnormal Nrf2 expression in DCs to suppress their maturation and T cell activation, in turn leading to immune escape [63]. Moreover, glioma cells actively recruit glioma-associated microglia/macrophages (GAMs) and induce M2 polarization [64]. M2-type GAMs produce numerous cytokines, interleukins, and growth factors that generate an immunosuppressive microenvironment and promote glioma cell growth, invasion and angiogenesis [62, 65]. Furthermore, infiltration by M2-polarized macrophages indicates an unfavorable prognosis in high-grade gliomas and confers an aggressive glioma subtype [66]. These changes give rise to a supportive environment, enrich for extracellular substrates, and maintain glioma growth or progression [62]. Since it is well established that tumor-infiltrating immune cells play a key role in tumor development, the mechanism by which ARL3 influences infiltrating immune cells in the glioma microenvironment should be investigated in future studies.

As a member of the ARF family, ARL3 is involved in regulating ciliary functions and lipid-modified proteins transport [20, 67]. Notably, ARL3 is a newly identified binding partner of STAT3 and enhances the phosphorylation and nuclear accumulation of STAT3 [26]. According to a recent study, ARL3 induces autophagy in HEK293T cells [68]. In the present study, we discovered that ARL3 expression was decreased in glioma samples and was associated with tumor grade. Furthermore, low expression of ARL3 was related to adverse outcomes and radiation and chemotherapy resistance in glioma. A nomogram with ARL3 was constructed and proven to accurately predict 3- or 5-year survival for glioma patients. Regarding biological function, we proved that ARL3 negatively regulates angiogenesis and influences immune cell infiltration into the glioma microenvironment. These findings complement the biological functions of ARL3 and may provide new options for the management of glioma.

## Conclusions

In conclusion, our study demonstrated that ARL3 expression was decreased in glioma samples and was associated with tumor grade. Furthermore, low ARL3 expression was related to adverse outcomes and radiation and chemotherapy resistance in glioma. We also constructed a nomogram with ARL3 to predict 3- or 5-survival for glioma patients. Regarding biological functions, we demonstrated that ARL3 was involved in angiogenesis and immune cell infiltration in the GBM immune microenvironment. Taken together, these results suggest a potential role of ARL3 as a prognostic marker and therapeutic target for glioma.

## Additional files

**Additional file 1: Table S1.** Prognostic values of differential expression genes of ARL members in GBM.

**Additional file 2: Table S2.** The clinical characteristics of 46 patients for ICH staining and survival analysis.

**Additional file 3: Figure S1.** ARL3 expression level was related to different response to radiation and chemotherapy in GBM. **Figure S2**. ROC curves and calibration curves for evaluating the accuracy of the nomogram in primary cohort.

**Additional file 4: Table S3.** Common genes related to ARL3 in the CGGA, TCGA and REMBRANDT.

**Additional file 5: Table S4.** ARL3-related biological terms in GBM via GSVA.

## Data Availability

The datasets used and/or analyzed during the current study are available from the corresponding author on reasonable request.

## Abbreviations

ARL3: ADP-ribosylation factor-like 3
ARL: ADP-ribosylation factor
GO: gene ontology
GSEA: gene set enrichment analysis
GSVA: gene set variation analysis
GBM: glioblastoma multiforme
GSCs: glioma stem cells
TCGA: The Cancer Genome Atlas
REMBRANDT: Repository for Molecular Brain Neoplasia Data
CGGA: Chinese Glioma Genome Atlas
ECM: extracellular matrix
PFS: progression free survival
OS: overall survival
MVD: microvascular density
DCs: dendritic cells
GAMs: glioma-associated microglia/macrophages
TCIA: The Cancer Immunome Atlas
TIMER: Tumor IMmune Estimation Resource
IVY GAP: The Ivy Glioblastoma Atlas Project

## References

[1] Lapointe S, Perry A, Butowski NA (2018). Primary brain tumours in adults. Lancet.

[2] Weller M, Butowski N, Tran DD, Recht LD, Lim M, Hirte H (2017). Rindopepimut with temozolomide for patients with newly diagnosed, EGFRvIII-expressing glioblastoma (ACT IV): a randomised, double-blind, international phase 3 trial. Lancet Oncol..

[3] Simonelli M, Persico P, Perrino M, Zucali PA, Navarria P, Pessina F (2018). Checkpoint inhibitors as treatment for malignant gliomas: “A long way to the top”. Cancer Treat Rev.

[4] Goldman DA, Hovinga K, Reiner AS, Esquenazi Y, Tabar V, Panageas KS (2018). The relationship between repeat resection and overall survival in patients with glioblastoma: a time-dependent analysis. J Neurosurg..

[5] Binder ZA, Thorne AH, Bakas S, Wileyto EP, Bilello M, Akbari H (2018). Epidermal growth factor receptor extracellular domain mutations in glioblastoma present opportunities for clinical imaging and therapeutic development. Cancer Cell.

[6] Ho IAW, Shim WSN (2017). Contribution of the microenvironmental niche to glioblastoma heterogeneity. Biomed Res Int.

[7] Fine HA (2015). New strategies in glioblastoma: exploiting the new biology. Clin Cancer Res.

[8] Gan HK, van den Bent M, Lassman AB, Reardon DA, Scott AM (2017). Antibody-drug conjugates in glioblastoma therapy: the right drugs to the right cells. Nat Rev Clin Oncol..

[9] Malo CS, Khadka RH, Ayasoufi K, Jin F, AbouChehade JE, Hansen MJ (2018). Immunomodulation mediated by anti-angiogenic therapy improves CD8 T cell immunity against experimental glioma. Front Oncol..

[10] Reiner DJ, Lundquist EA (2018). Small GTPases. WormBook..

[11] Li X, Liu S, Fang X, He C, Hu X (2019). The mechanisms of DIRAS family members in role of tumor suppressor. J Cell Physiol..

[12] Bueno A, Morilla I, Diez D, Moya-Garcia AA, Lozano J, Ranea JA (2016). Exploring the interactions of the RAS family in the human protein network and their potential implications in RAS-directed therapies. Oncotarget..

[13] Kahn RA, Volpicelli-Daley L, Bowzard B, Shrivastava-Ranjan P, Li Y, Zhou C (2005). Arf family GTPases: roles in membrane traffic and microtubule dynamics. Biochem Soc Trans.

[14] Casalou C, Faustino A, Barral DC (2016). Arf proteins in cancer cell migration. Small GTPases..

[15] Platet N, Hinkel I, Richert L, Murdamoothoo D, Moufok-Sadoun A, Vanier M (2017). The tumor suppressor CDX2 opposes pro-metastatic biomechanical modifications of colon cancer cells through organization of the actin cytoskeleton. Cancer Lett.

[16] Franzetti GA, Laud-Duval K, van der Ent W, Brisac A, Irondelle M, Aubert S (2017). Cell-to-cell heterogeneity of EWSR1-FLI1 activity determines proliferation/migration choices in Ewing sarcoma cells. Oncogene.

[17] Zhou C, Cunningham L, Marcus AI, Li Y, Kahn RA (2006). Arl2 and Arl3 regulate different microtubule-dependent processes. Mol Biol Cell.

[18] Jin M, Yamada M, Arai Y, Nagai T, Hirotsune S (2014). Arl3 and LC8 regulate dissociation of dynactin from dynein. Nat Commun..

[19] Ismail SA, Chen YX, Rusinova A, Chandra A, Bierbaum M, Gremer L (2011). Arl2-GTP and Arl3-GTP regulate a GDI-like transport system for farnesylated cargo. Nat Chem Biol.

[20] Fansa EK, Wittinghofer A (2016). Sorting of lipidated cargo by the Arl2/Arl3 system. Small GTPases..

[21] Schrick JJ, Vogel P, Abuin A, Hampton B, Rice DS (2006). ADP-ribosylation factor-like 3 is involved in kidney and photoreceptor development. Am J Pathol.

[22] Lokaj M, Kosling SK, Koerner C, Lange SM, van Beersum SE, van Reeuwijk J (2015). The interaction of CCDC104/BARTL1 with Arl3 and implications for ciliary function. Structure..

[23] Li T, Fan J, Wang B, Traugh N, Chen Q, Liu JS (2017). TIMER: a web server for comprehensive analysis of tumor-infiltrating immune cells. Cancer Res.

[24] Wu J, Zhao W, Zhou B, Su Z, Gu X, Zhou Z (2018). TSNAdb: a database for tumor-specific neoantigens from immunogenomics data analysis. Genomics Proteom Bioinform..

[25] Charoentong P, Finotello F, Angelova M, Mayer C, Efremova M, Rieder D (2017). Pan-cancer immunogenomic analyses reveal genotype-immunophenotype relationships and predictors of response to checkpoint blockade. Cell Rep..

[26] Togi S, Muromoto R, Hirashima K, Kitai Y, Okayama T, Ikeda O (2016). A New STAT3-binding partner, ARL3, enhances the phosphorylation and nuclear accumulation of STAT3. J Biol Chem.

[27] Cheng P, Phillips E, Kim SH, Taylor D, Hielscher T, Puccio L (2015). Kinome-wide shRNA screen identifies the receptor tyrosine kinase AXL as a key regulator for mesenchymal glioblastoma stem-like cells. Stem Cell Reports..

[28] Wang Y, Guan G, Cheng W, Jiang Y, Shan F, Wu A (2018). ARL2 overexpression inhibits glioma proliferation and tumorigenicity via down-regulating AXL. BMC Cancer..

[29] Cheng P, Wang J, Waghmare I, Sartini S, Coviello V, Zhang Z (2016). FOXD1-ALDH1A3 signaling is a determinant for the self-renewal and tumorigenicity of mesenchymal glioma stem cells. Cancer Res.

[30] Carbon S, Ireland A, Mungall CJ, Shu S, Marshall B, Lewis S (2009). AmiGO: online access to ontology and annotation data. Bioinformatics.

[31] Mi H, Huang X, Muruganujan A, Tang H, Mills C, Kang D (2017). PANTHER version 11: expanded annotation data from gene ontology and reactome pathways, and data analysis tool enhancements. Nucleic Acids Res.

[32] da Huang W, Sherman BT, Lempicki RA (2009). Systematic and integrative analysis of large gene lists using DAVID bioinformatics resources. Nat Protoc.

[33] Hanzelmann S, Castelo R, Guinney J (2013). GSVA: gene set variation analysis for microarray and RNA-seq data. BMC Bioinform.

[34] Subramanian A, Tamayo P, Mootha VK, Mukherjee S, Ebert BL, Gillette MA (2005). Gene set enrichment analysis: a knowledge-based approach for interpreting genome-wide expression profiles. Proc Natl Acad Sci USA.

[35] Qian J, Luo F, Yang J, Liu J, Liu R, Wang L (2018). TLR2 promotes glioma immune evasion by downregulating MHC class II molecules in microglia. Cancer Immunol Res.

[36] Wang Q, Hu B, Hu X, Kim H, Squatrito M, Scarpace L (2017). Tumor evolution of glioma-intrinsic gene expression subtypes associates with immunological changes in the microenvironment. Cancer Cell.

[37] Stone GW, Maehara A, Lansky AJ, de Bruyne B, Cristea E, Mintz GS (2011). A prospective natural-history study of coronary atherosclerosis. N Engl J Med.

[38] Mur P, RodriguezdeLope A, Diaz-Crespo FJ, Hernandez-Iglesias T, Ribalta T, Fiano C (2015). Impact on prognosis of the regional distribution of MGMT methylation with respect to the CpG island methylator phenotype and age in glioma patients. J Neurooncol..

[39] Rasmussen BK, Hansen S, Laursen RJ, Kosteljanetz M, Schultz H, Norgard BM (2017). Epidemiology of glioma: clinical characteristics, symptoms, and predictors of glioma patients grade I–IV in the Danish Neuro-Oncology Registry. J Neurooncol.

[40] Ostrom QT, Coleman W, Huang W, Rubin JB, Lathia JD, Berens ME (2019). Sex-specific gene and pathway modeling of inherited glioma risk. Neuro Oncol..

[41] Kingsmore KM, Vaccari A, Abler D, Cui SX, Epstein FH, Rockne RC (2018). MRI analysis to map interstitial flow in the brain tumor microenvironment. APL Bioeng..

[42] Najafi M, Goradel NH, Farhood B, Salehi E, Solhjoo S, Toolee H (2019). Tumor microenvironment: Interactions and therapy. J Cell Physiol..

[43] Loveson KF, Fillmore HL (2018). Intersection of brain development and paediatric diffuse midline gliomas: potential role of microenvironment in tumour growth. Brain Sci..

[44] Ma X, Li Z, Li T, Zhu L, Li Z, Tian N (2017). Long non-coding RNA HOTAIR enhances angiogenesis by induction of VEGFA expression in glioma cells and transmission to endothelial cells via glioma cell derived-extracellular vesicles. Am J Transl Res..

[45] Liu Y, Carson-Walter EB, Cooper A, Winans BN, Johnson MD, Walter KA (2010). Vascular gene expression patterns are conserved in primary and metastatic brain tumors. J Neurooncol.

[46] Onishi M, Ichikawa T, Kurozumi K, Inoue S, Maruo T, Otani Y (2015). Annexin A2 regulates angiogenesis and invasion phenotypes of malignant glioma. Brain Tumor Pathol..

[47] Domingues P, Gonzalez-Tablas M, Otero A, Pascual D, Miranda D, Ruiz L (2016). Tumor infiltrating immune cells in gliomas and meningiomas. Brain Behav Immun.

[48] Ghosh D, Nandi S, Bhattacharjee S (2018). Combination therapy to checkmate glioblastoma: clinical challenges and advances. Clin Transl Med..

[49] Jain KK (2018). A critical overview of targeted therapies for glioblastoma. Front Oncol..

[50] Friedmann-Morvinski D (2014). Glioblastoma heterogeneity and cancer cell plasticity. Crit Rev Oncog.

[51] Wang Y, Li J, Xia Y, Gong R, Wang K, Yan Z (2013). Prognostic nomogram for intrahepatic cholangiocarcinoma after partial hepatectomy. J Clin Oncol.

[52] Wang C, Yang C, Wang W, Xia B, Li K, Sun F (2018). A prognostic nomogram for cervical cancer after surgery from SEER database. J Cancer..

[53] Sasaki T, Fukai J, Kodama Y, Hirose T, Okita Y, Moriuchi S (2018). Characteristics and outcomes of elderly patients with diffuse gliomas: a multi-institutional cohort study by Kansai Molecular Diagnosis Network for CNS Tumors. J Neurooncol.

[54] Gittleman H, Lim D, Kattan MW, Chakravarti A, Gilbert MR, Lassman AB (2017). An independently validated nomogram for individualized estimation of survival among patients with newly diagnosed glioblastoma: NRG Oncology RTOG 0525 and 0825. Neuro Oncol..

[55] Turkalp Z, Karamchandani J, Das S (2014). IDH mutation in glioma: new insights and promises for the future. JAMA Neurol..

[56] Weller M, Wick W, Aldape K, Brada M, Berger M, Pfister SM (2015). Glioma. Nat Rev Dis Primers..

[57] Tan Z, Chen K, Wu W, Zhou Y, Zhu J, Wu G (2018). Overexpression of HOXC10 promotes angiogenesis in human glioma via interaction with PRMT5 and upregulation of VEGFA expression. Theranostics..

[58] Zhu C, Kros JM, Cheng C, Mustafa D (2017). The contribution of tumor-associated macrophages in glioma neo-angiogenesis and implications for anti-angiogenic strategies. Neuro Oncol..

[59] Winkler F, Osswald M, Wick W (2018). Anti-angiogenics: their role in the treatment of glioblastoma. Oncol Res Treat..

[60] Touat M, Idbaih A, Sanson M, Ligon KL (2017). Glioblastoma targeted therapy: updated approaches from recent biological insights. Ann Oncol.

[61] Wang N, Jain RK, Batchelor TT (2017). New directions in anti-angiogenic therapy for glioblastoma. Neurotherapeutics..

[62] Gieryng A, Pszczolkowska D, Walentynowicz KA, Rajan WD, Kaminska B (2017). Immune microenvironment of gliomas. Lab Invest.

[63] Wang J, Liu P, Xin S, Wang Z, Li J (2017). Nrf2 suppresses the function of dendritic cells to facilitate the immune escape of glioma cells. Exp Cell Res.

[64] Roesch S, Rapp C, Dettling S, Herold-Mende C (2018). When immune cells turn bad-tumor-associated microglia/macrophages in glioma. Int J Mol Sci..

[65] Xu Y, Liao C, Liu R, Liu J, Chen Z, Zhao H (2019). IRGM promotes glioma M2 macrophage polarization through p62/TRAF6/NF-kappaB pathway mediated IL-8 production. Cell Biol Int..

[66] Sorensen MD, Dahlrot RH, Boldt HB, Hansen S, Kristensen BW (2018). Tumour-associated microglia/macrophages predict poor prognosis in high-grade gliomas and correlate with an aggressive tumour subtype. Neuropathol Appl Neurobiol.

[67] Zhang Q, Hu J, Ling K (2013). Molecular views of Arf-like small GTPases in cilia and ciliopathies. Exp Cell Res.

[68] Luo G, Sun Y, Feng R, Zhao Q, Wen T (2018). ARL3 subcellular localization and its suspected role in autophagy. Biochimie.

