# Supplementary material for: ARL3 is downregulated and acts as a prognostic biomarker in glioma
Source: J Transl Med. 2019 Jun 24;17:210. doi: 10.1186/s12967-019-1914-3 (PMC6591946; doi:10.1186/s12967-019-1914-3)
Supplement: Supplementary file 1 — Additional file 1: Table S1. Prognostic values of differential expression genes of ARL members in GBM. [file 12967_2019_1914_MOESM1_ESM.docx]

**Additional file 1: Table S1. Prognostic values of differential expression genes of ARL members in GBM**

| Variables | Univariate analysis | |
| --- | --- | --- |
|  | **HR** | **P value** |
| ARL2 | 0.872 | 0.327 |
| ARL3 | 0.674 | 0.016 |
| ARL4A | 1.285 | 0.002 |
| ARL4C | 1.20 | 0.018 |
| ARL5A | 1.246 | 0.202 |
| ARL6 | 1.408 | 0.062 |
| ARL8A | 1.168 | 0.517 |
| ARL8B | 1.372 | 0.070 |
| ARL11 | 1.629 | 0.010 |
| ARL13B | 1.123 | 0.415 |
| ARL15 | 1.019 | 0.908 |
| ARL16 | 0.750 | 0.143 |
