# Supplementary material for: ARL3 is downregulated and acts as a prognostic biomarker in glioma
Source: J Transl Med. 2019 Jun 24;17:210. doi: 10.1186/s12967-019-1914-3 (PMC6591946; doi:10.1186/s12967-019-1914-3)
Supplement: Supplementary file 2 — Additional file 2: Table S2. The clinical characteristics of 46 patients for ICH staining and survival analysis. [file 12967_2019_1914_MOESM2_ESM.docx]

**Additional file 2: Table S2. The clinical characteristics of 46 patients for ICH staining and survival analysis**

| Characteristic | ARL3 expression level | |
| --- | --- | --- |
|  | **Low**, n=29 | **High**, n=17 |
| Age, years  Mean, range | 47.2 (25-79) | 41.6 (28-80) |
| Gender  Male  Female | 17  12 | 10  7 |
| WHO grade  Grade II  Grade III  Grade IV | 1  10  18 | 4  7  6 |
