# Supplementary material for: ARL3 is downregulated and acts as a prognostic biomarker in glioma
Source: J Transl Med. 2019 Jun 24;17:210. doi: 10.1186/s12967-019-1914-3 (PMC6591946; doi:10.1186/s12967-019-1914-3)

**ARL3 is down-regulated and acts as a prognostic biomarker in glioma**

Yulin Wang^1^, Weijiang Zhao^2^, Xin Liu^3^, Gefei Guan^4^, Minghua Zhuang^1^*

^1^ Department of Neurosurgery, The First Affiliated Hospital of Shantou University Medical College, Shantou, Guangdong 515041, China

^2^ Center for Neuroscience, Shantou University Medical College, Shantou, Guangdong 515041, China

^3^ Department of stomatology, The First Affiliated Hospital of Shantou University Medical College, Shantou, Guangdong 515041, China

^4^ Department of Neurosurgery, The First Hospital of China Medical University, Shenyang, Liaoning 110001, China

**Additional file 3: Figures**

**Additional file 3: Figure S1 ARL3 expression suggested differential responses to radiation and chemotherapy in GBM patients**

1. Data from TCGA indicated that the GBM patients receiving radiotherapy in high ARL3 group had a favorable prognosis compared with low group (HG-UG 133A; low, n = 216; high, n = 162; *P* = 0.0067, with log-rank test).
2. Data from TCGA revealed that high ARL3 expression exhibited better curative effect of chemotherapy than low ARL3 expression group in GBM patients (HG-UG 133A; low, n = 204; high, n = 151; *P* = 0.0151, with log-rank test).


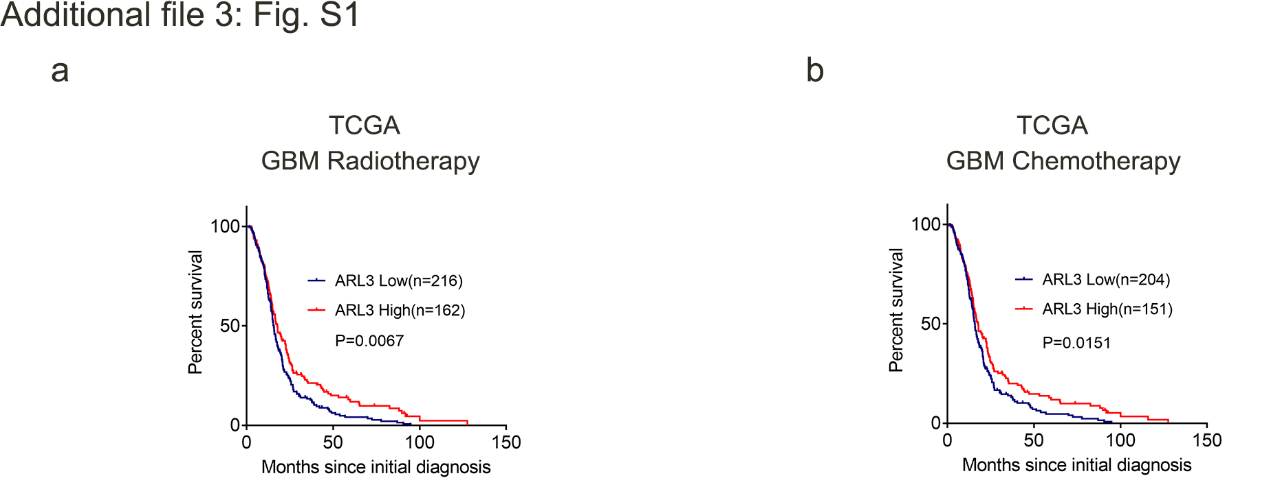


**Additional file 3: Figure S2 ROC curve and calibration curve were used to evaluate the accuracy of the nomogram in primary cohort**

1. ROC curve was applied to evaluate the accuracy of 3 and 5-year survival in the primary cohort. The AUC of ROC in the nomogram for 3 or 5 year-survival were 0.932 and 0.898 in primary cohort, respectively.

(b) The calibration plot for the probability of survival at 3-year showed an optimal agreement between the prediction and observation in the primary cohort.

(c) The calibration plot for the probability of survival at 5-year showed a good coincidence between the prediction and observation in the primary cohort.


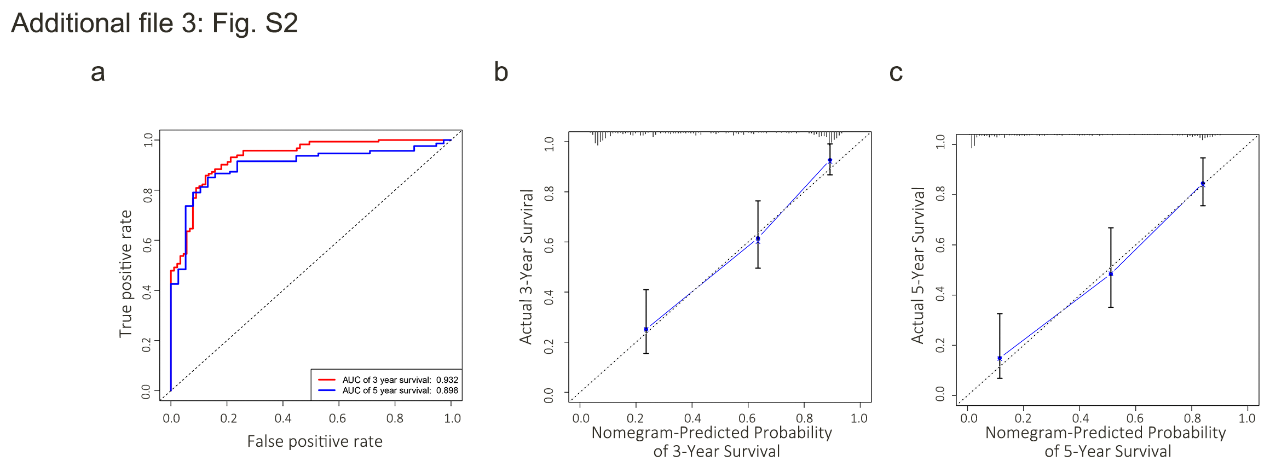

Supplement: Supplementary file 3 — Additional file 3: Figure S1. ARL3 expression level was related to different response to radiation and chemotherapy in GBM. Figure S2. ROC curves and calibration curves for evaluating the accuracy of the nomogram in primary cohort. [file 12967_2019_1914_MOESM3_ESM.docx]
